# Compensation Method of Natural Head Movement for Gaze Tracking System Using an Ultrasonic Sensor for Distance Measurement

**DOI:** 10.3390/s16010110

**Published:** 2016-01-16

**Authors:** Dongwook Jung, Jong Man Lee, Su Yeong Gwon, Weiyuan Pan, Hyeon Chang Lee, Kang Ryoung Park, Hyun-Cheol Kim

**Affiliations:** 1 Division of Electronics and Electrical Engineering, Dongguk University, 30, Pildong-ro 1-gil, Jung-gu, Seoul 100-715, Korea; jung4759@gmail.com (D.J.); jmlee1019@dongguk.edu (J.M.L.); adeptkang@naver.com (S.Y.G); westlaopan90@gmail.com (W.P.); leehc@dongguk.edu (H.C.L); 2 Electronics and Telecommunications Research Institute, 218 Gajeong-ro, Yuseong-gu, Daejeon 305-700, Korea; kimhc@etri.re.kr

**Keywords:** gaze tracking system, compensation of head movements, ultrasonic sensor, natural head movement

## Abstract

Most gaze tracking systems are based on the pupil center corneal reflection (PCCR) method using near infrared (NIR) illuminators. One advantage of the PCCR method is the high accuracy it achieves in gaze tracking because it compensates for the pupil center position based on the relative position of corneal specular reflection (SR). However, the PCCR method only works for user head movements within a limited range, and its performance is degraded by the natural movement of the user’s head. To overcome this problem, we propose a gaze tracking method using an ultrasonic sensor that is robust to the natural head movement of users. Experimental results demonstrate that with our compensation method the gaze tracking system is more robust to natural head movements compared to other systems without our method and commercial systems.

## 1. Introduction

Gaze tracking is a technology to find out where a user is looking. It has been widely used in various applications such as neuroscience, psychology, industrial engineering, human factors, marketing, advertising, and computer interfaces [1]. There has been a lot of research on gaze tracking based on the movement of face and eyes [2], and on the adoption of gaze tracking technology in natural input devices to replace the conventional keyboard and mouse [3,4,5,6]. With the increased adoption of gaze tracking technology in various fields, studies aiming to improve gaze tracking accuracy have been progressing actively. Most of the known gaze tracking systems use the pupil center corneal reflection (PCCR) method [7,8,9,10,11,12,13,14,15,16,17,18,19,20,21,22]. In this method, the position of the corneal specular reflection (SR) determined by a near infrared (NIR) illuminator is regarded as a reference. The final gaze position on the screen is calculated using the vector from the corneal SR to the pupil center (PC), and the matrix defining the relationship between the four corner positions of the screen and the corresponding four vectors obtained during calibration. However, this matrix is obtained at one specific Z-distance between the user and the screen. Therefore, if a user’s head moves after calibration, the matrix is considered incorrect for calculating the gaze position, which increases the consequent gaze detection error.

To overcome this problem, there has been research focusing on compensating for the user’s head movement. We can categorize the past research into two types: multiple camera-based and single camera-based methods. In multiple camera-based methods [15,16,17,18,23], two or more cameras are used so that more 2D or 3D information about the user’s eye position, by which the movement of user’s head can be compensated, can be acquired, compared to single camera-based methods. In previous research [15,16,23], two cameras are used as a stereo vision system to acquire the 3D eye position more accurately. A stereo camera can simulate human binocular vision; therefore, it can acquire 3D information about the user’s eye. In other research [17,18], two static wide-view cameras mounted on the left and the right sides of the display are used as head cameras. The images acquired by these two cameras are used for analyzing the depth information of users’ eyes. Another narrow-view camera (eye camera) mounted on a pan-tilt unit is placed under the display for capturing one of the two eyes of the user. In order to measure the gaze direction precisely, the eye is ‘zoomed-in’ in the image. The viewing direction of the narrow-view camera is controlled by the pan and tilt unit. However, using multiple cameras increases the size and cost of the system, and additional calibration among cameras becomes necessary.

In the single camera-based methods, multiple illuminators are used [19,20]. By using multiple SRs from multiple illuminators, the movement of a user’s head after calibration can be compensated. In previous research [19], three NIR light emitting diode (LED) illuminators are used. A ring of NIR LEDs attached around the camera lens is used to generate a bright pupil image. Two NIR illuminators located beside the computer screen produce the dark pupil image, which contains the dual corneal SRs. The ring of NIR LEDs (generating the bright pupil image) and two NIR illuminators (producing the dark pupil image) are alternately turned on and off. For example, the first eye image (the bright pupil image) is captured while the ring of NIR LEDs is turned on and two NIR illuminators are turned off. Then, the second image (the dark pupil image) is captured while the ring of NIR LEDs is turned off and two NIR illuminators are turned on. This procedure of switching between turning on/off is repeated at fast speed, from which the eye movement in the two images (the bright and dark pupil images) can be minimized. Then, subtracting the dark pupil image from the bright pupil image enhances the pupil contour, which makes it easier to detect the accurate pupil area. In [20], dual NIR illuminators are used in the proposed gaze tracking system. The authors define the center position of two corneal SRs as a corneal SR center. Based on the positional information of corneal SRs and pupil, they compensate the head movement for accurate gaze detection.

However, multiple corneal SRs can appear near the pupil boundary, which can decrease the accuracy of pupil center detection and consequent gaze tracking compared to the methods based on a single illuminator. In addition, more reflections usually exist on the surface of glasses in case of user wearing glasses, which can make it difficult to detect the correct corneal SR and pupil center. To overcome these problems, we propose a new gaze tracking method based on single camera and single illuminator with a small-sized ultrasonic sensor, which is robust to the natural movement of user’s head. Because the ultrasonic sensor can successively measure the Z-distance of user independently from the operation of gaze tracking camera, it cannot give any burden to our gaze tracking system. Our research is novel in the following four ways compared to previous research.

- We perform mathematical analyses of the change of relative positions between the PC and corneal SR according to various head movements such as rotations and translations based on X-, Y-, and Z-axes. Based on these analyses, we find that only the head movement (causing the change of Z-distance between the camera and user’s eye) can affect the accuracy of gaze detection.
- Based on this result, we measure the change of Z-distance of user’s eye using a small sized ultrasonic sensor. The ultrasonic sensor-based method does not require complicated camera calibration and it measures the Z-distance much faster than stereo cameras.
- The accuracy of measured Z-distance by the ultrasonic sensor can be greatly enhanced by using the change of inter-distance of two eyes in the image, and the actual inter-distance in 3D space is measured by the ultrasonic sensor and the detected positions of two pupil centers in the image during the procedure of user calibration.
- By using the additional information of the change of movement of both eyes in X-axis of the image, we can discriminate the head rotation from the head translation in the direction of Z-axis.

The remainder of this paper is organized as follows: in Section 2, our proposed gaze tracking method that is robust to head movement is described, in Section 3, the experimental results and analyses are provided, and in Section 4, we provide concluding remarks and directions for future studies in the area.

## 2. Proposed Method for Compensating User’s Head Movement

In our gaze tracking system, we acquire the Z-distance data using an ultrasonic sensor as well as using the actual inter-distance of two pupil centers obtained during a user calibration stage. Then, we measure the Z-distance of user’s eye from the camera based on the actual inter-distance of two pupil centers during user calibration stage. In the testing stage, our gaze tracking system calculates the difference between the Z-distance of user’s eye when capturing the current image and that obtained during user calibration. If this difference is not greater than a threshold, the user’s head movement does not need to be compensated, and our system calculates the gaze position without compensation. If this difference is greater than a threshold, our system checks whether a case of head rotation (yaw) based on the vertical axis has occurred. If this case has not occurred, our system compensates the user’s head movement and calculates the gaze position. If it has occurred the user’s head movement does not need to be compensated, and our system calculates the gaze position without compensation.

### 2.1. Gaze Tracking Algorithm

Our gaze tracking algorithm is based on the PCCR method [7,21], which is the most commonly used method using 2-D mapping-based approach for gaze tracking. Details of our gaze tracking method are provided below with explanations.

**Figure 1:**
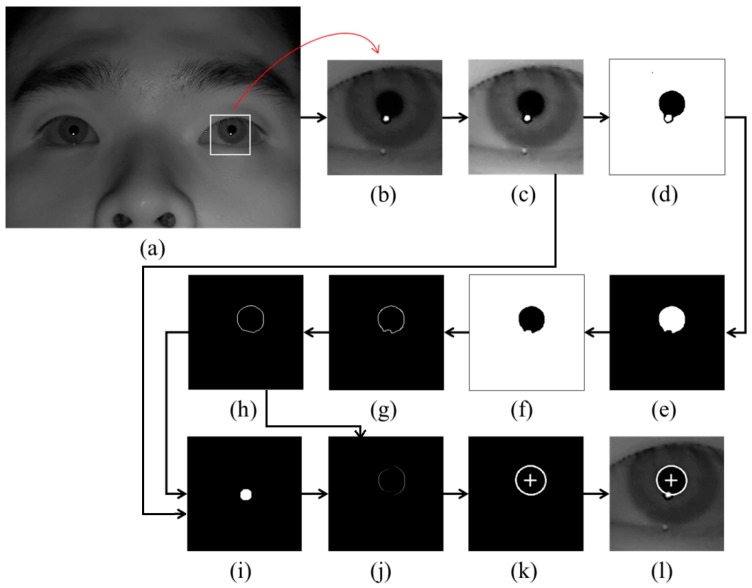
Examples of pupil center and boundary detection: (**a**) rough position of corneal SR is located in the original image (**b**) region for detecting pupil area is defined. Result images by (**c**) histogram stretching, (**d**) image binarization, (**e**) morphological operation, (**f**) component labeling, (**g**) canny edge detection, (**h**) convex hull method, (**i**) image binarization of (**c**), (**j**) subtracting the overlapped parts of (**h**) and (**i**) from (**h**), (**k**) ellipse-fitting method, and (**l**) final detection result of pupil center and boundary.

As the first step, the rough position of the corneal SR is identified by finding the bright pixels in the captured image. Then, the region for detecting the pupil area is defined based on the identified position of the corneal SR as shown in Figure 1b. In order to make the pupil boundary more distinctive, histogram stretching is applied to this region (Figure 1c), and binarized image is obtained because pupil area is usually darker than the surrounding regions as shown in Figure 1d. Then, only the pupil region is left in the image by excluding the corneal SR and other noise regions through the procedures of morphological operation and component labeling as shown in Figure 1e,f. Pupil boundary points are located by canny edge detector as shown in Figure 1g, and the damaged part on the pupil boundary by the corneal SR can be compensated (Figure 1h) through the convex hull method procedure [24,25,26]. A binarized image of Figure 1c is obtained as shown in Figure 1i. Then, the overlapped region of Figure 1h,i is removed from the image of Figure 1h as shown in Figure 1j. With Figure 1j, sn accurate pupil boundary can be detected by excluding the corneal SR. With the remaining points on the pupil boundary, the accurate pupil boundary is detected by ellipse-fitting method as shown in Figure 1k. The results of pupil boundary and center detection are shown in Figure 1l.

**Figure 2:**
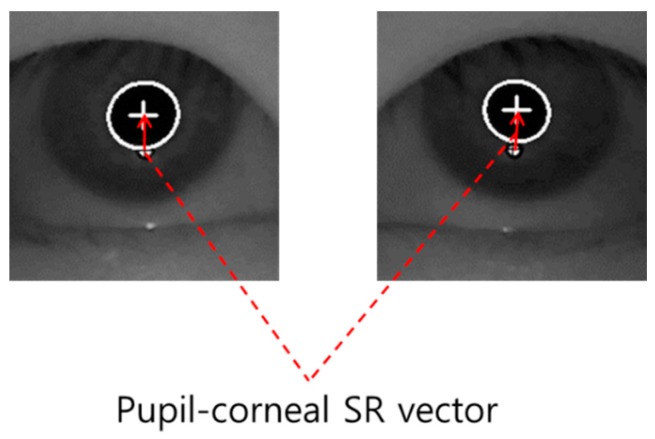
Example of pupil-corneal SR vector.

When the pupil center and boundary are detected as shown in Figure 1l, our gaze detection algorithm defines the searching area for detecting the accurate position of corneal SR. Within this area, the accurate center of corneal SR is detected through the image binarization process and by calculating the geometric center position. Based on the detected centers of pupil and corneal SR, the pupil-corneal SR vector can be obtained as shown in Figure 2.

**Figure 3:**
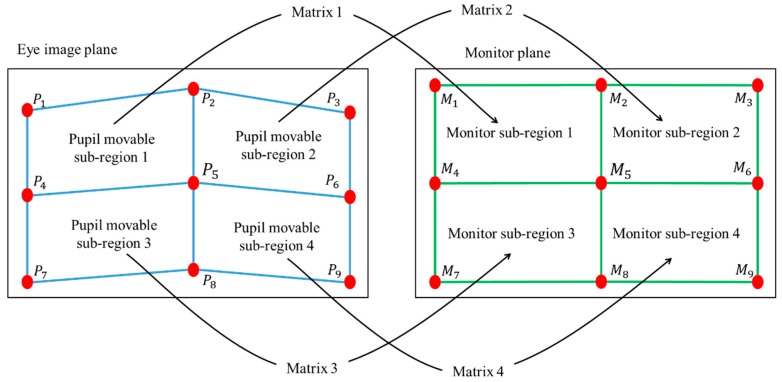
Relationship between pupil movable sub-regions and monitor sub-regions.

Based on the pupil-corneal SR vector, the user’s gaze position is calculated using a geometric transform method as follows [7,21]. Pupil-corneal SR vector is used for obtaining the mapping relationship between the pupil movable sub-region and monitor sub-region as shown in Figure 3. In our gaze tracking system, each user should look at nine reference points (*M*_1_, *M*_2_, … *M*_9_ of Figure 3) on the monitor during the user calibration stage. From that, nine pupil centers in the images are acquired, and nine pupil-corneal SR vectors are consequently obtained. In order to compensate the corneal SR movement caused by the head movement, all the starting positions of nine pupil-corneal SR vectors are superimposed, and the compensated nine pupil centers (*P*_1_, *P*_2_, … *P*_9_ of Figure 3) in the images are acquired. We include detailed explanations about the procedure for compensating the pupil centers as follows. For example, the positions of the corneal SR and pupil center are assumed to be (120, 120) and (110, 110), respectively, in the first image. In addition, those of the corneal SR and pupil center are assumed to be (105, 134) and (130, 90), respectively, in the second image. Then, the amount of movement between the two corneal SR positions in the first and second images becomes −15 (105−120) and +14 (134−120) on the *x*- and *y*-axis, respectively. Therefore, if we attempt to set the position of corneal SR in the first image (120, 120) to be the same as that in the second image (105, 134) (the vectors of corneal SR coincide), the position of pupil center position in the first image (110, 110) is changed to be (95, 124) considering the amount of movement (−15, +14). This new position of (95, 124) is the compensated pupil center, which is used for calculating gaze position instead of the original position of pupil center (110, 110).

Then, four pupil movable sub-regions and monitor sub-regions are defined, and a geometric transform matrix that defines each pair of pupil movable sub-regions and monitor sub-regions is obtained as shown in Figure 3 and Equation (1). 

In Equation (1), *n* is 1, 2, 4, and 5. The four points of (*P*_(n+i)x_, *P*_(n+i)y_) and (*M*_(n+i)x_, *M*_(n+i)y_) (i is 0, 1, 3, and 4) represent the X and Y positions of compensated pupil center (in eye image) and reference point (on the monitor), respectively. From Equation (1), we can obtain eight unknown parameters (*a, b, c ... h*) using matrix inversion:

(1) $$\left[\begin{array}{cccc} M_{nx} & M_{\left(n+1\right)x} & M_{\left(n+3\right)x} & M_{\left(n+4\right)x}\\ M_{ny} & M_{\left(n+1\right)y} & M_{\left(n+3\right)y} & M_{\left(n+4\right)y}\\ 0 & 0 & 0 & 0\\ 0 & 0 & 0 & 0 \end{array}\right]=\left[\begin{array}{cccc} a & b & c & d\\ e & f & g & h\\ 0 & 0 & 0 & 0\\ 0 & 0 & 0 & 0 \end{array}\right]\left[\begin{array}{cccc} P_{nx} & P_{\left(n+1\right)x} & P_{\left(n+3\right)x} & P_{\left(n+4\right)x}\\ P_{ny} & P_{\left(n+1\right)y} & P_{\left(n+3\right)y} & P_{\left(n+4\right)y}\\ P_{nx}P_{ny} & P_{\left(n+1\right)x}P_{\left(n+1\right)y} & P_{\left(n+3\right)x}P_{\left(n+3\right)y} & P_{\left(n+4\right)x}P_{\left(n+4\right)y}\\ 1 & 1 & 1 & 1 \end{array}\right]$$

In general, the shape transform from one quadrangle to the other can be mathematically defined by using several unknown parameters as shown in [27]. If one quadrangle is changed to the other only by in-plane rotation and translation (x-axis and y-axis), the transform matrix can be defined by using three unknown parameters. If one quadrangle is changed to the other only by in-plane rotation, translation (x-axis and y-axis), and scaling, the transform matrix can be defined by using four unknown parameters. In case that one quadrangle is changed to the other only by in-plane rotation, translation (x-axis and y-axis), scaling, and parallel inclination (x-axis and y-axis), the transform matrix can be defined by using six unknown parameters. In the last case, if one quadrangle is changed to the other by in-plane rotation, translation (x-axis and y-axis), scaling, parallel inclination (x-axis and y-axis), and distortion (x-axis and y-axis), the transform matrix can be defined by using eight unknown parameters [27]. Because the shape transform from one pupil movable sub-region of Figure 3 to one monitor sub-region of Figure 3 can include the various factors of the last case, we define the transform matrix by using eight unknown parameters.

Once we obtain the parameters (*a, b, c ... h*), we can calculate where the user is staring at on the monitor (*M^’^_x_* and *M^’^_y_* of Equation (2)) using the compensated pupil center in a current frame (*P^’^_x_* and *P^’^_y_* Equation (2)):

(2) $$\left[\begin{array}{c} \begin{array}{c} M\prime_{x}\\ M\prime_{y} \end{array}\\ \begin{array}{c} 0\\ 0 \end{array} \end{array}\right]=\left[\begin{array}{c} \begin{array}{cc} a & b\\ e & f \end{array}\;\;\;\;\begin{array}{cc} c & d\\ g & h \end{array}\\ \begin{array}{cc} 0 & 0\\ 0 & 0 \end{array}\;\;\;\;\begin{array}{cc} 0 & 0\\ 0 & 0 \end{array} \end{array}\right]\left[\begin{array}{c} \begin{array}{c} P\prime_{x}\\ P\prime_{y} \end{array}\\ \begin{array}{c} P\prime_{x}P\prime_{y}\\ 1 \end{array} \end{array}\right]$$

### 2.2. Analyses of the Change of Pupil-Corneal SR Vectors According to Head Movements

**Figure 4:**
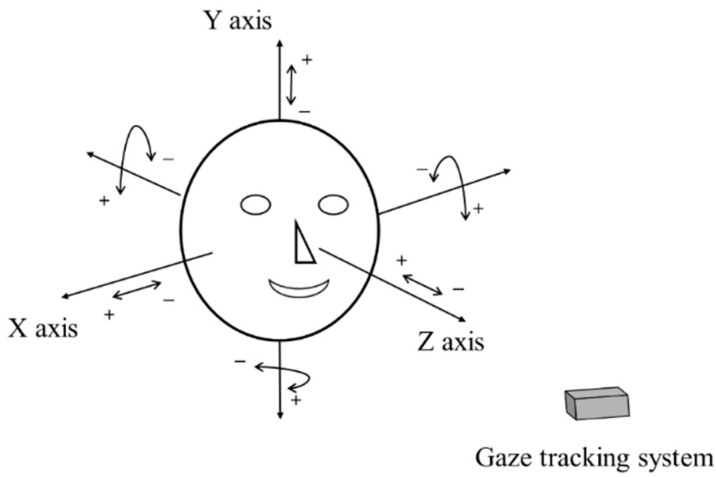
Definition of head rotation and translation.

The geometric transform matrix of Equation (2) is obtained at one Z-distance between the user and the screen. Therefore, if the user’s head is moved after the calibration, the matrix is not correct for calculating the gaze position, which increases the consequent gaze detection error. To overcome this problem, we propose the method of compensating user’s head movement using an ultrasonic sensor. Before explaining our compensation method in detail, we define the user’s head movements according to translation and rotation movement based on the X-, Y-, and Z-axes, respectively, in 3D space, as shown in Figure 4. In addition, we define the symbols representing the head movements in Table 1.

**Table 1:** Definition of the symbol representing the head movements.

Head Movements	X Axis	Y Axis	Z Axis
Translation	T_X{+, –}	T_Y{+, –}	T_Z{+, –}
Rotation	R_X{+, –} (Pitch)	R_Y{+, –} (Yaw)	R_Z{+, –} (Roll)

**Figure 5:**
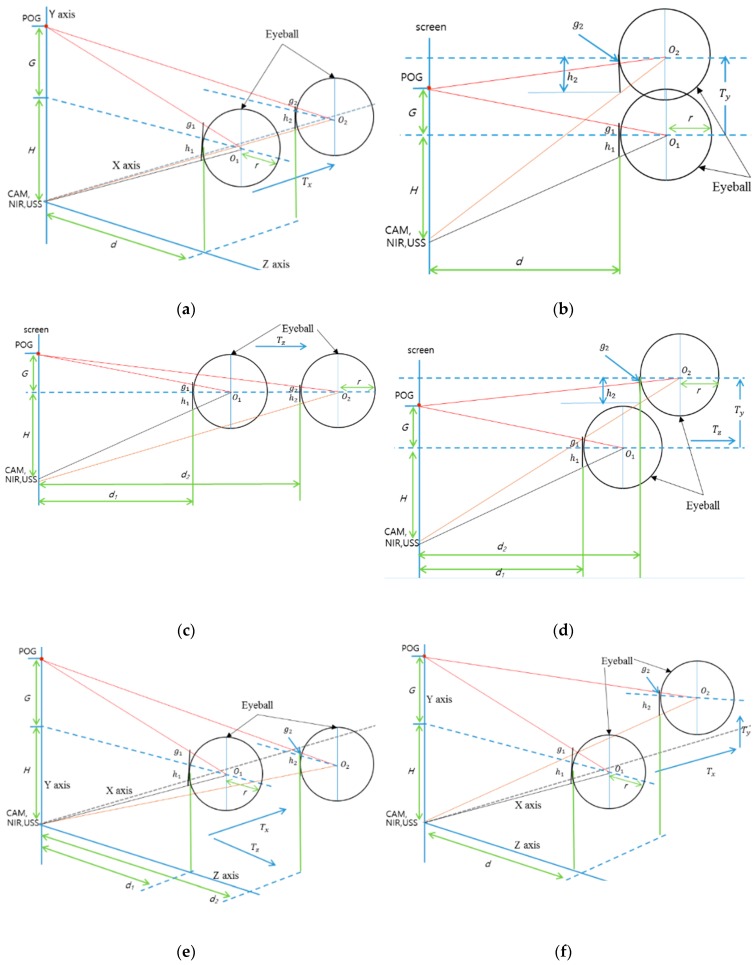
Conceptual diagrams of the changes of pupil-corneal SR vector according to head movements: (**a**) T_X movement, (**b**) T_Y movement (side view), (**c**) T_Z movement (side view), (**d**) R_X movement (compound of T_Y and T_Z, side view), (**e**) R_Y movement (compound of T_X and T_Z), and (**f**) R_Z movement (compound of T_X and T_Y).

Considering the definition of head rotation and translation as shown in Figure 4 and Table 1, we analyze the change of pupil-corneal SR vectors according to the head movements. In Figure 5, the circular shape represents a user’s eyeball, and *r* is the radius of the eyeball based on Gullstrand’s eye model [28]. POG means a point of gaze, and it is connected by user’s pupil center and eyeball’s center (*O*_1_ or *O*_2_). *G* represents the distance from POG to the Y position of eyeball’s center*. H* shows the distance from camera (CAM)/near infrared (NIR) illuminator/ultrasonic sensor (USS) to the Y position of eyeball’s center. *d* represents the distance from X-Y plane to the nearest surface of eyeball. *g* (*g*_1_ or *g*_2_) shows the distance from eyeball’s surface center to the line passing through POG and eyeball’s center. *h* (*h*_1_ or *h*_2_) represents the distance from the eyeball’s surface center to the line passing through camera/NIR illuminator/ultrasonic sensor, and eyeball’s center. The line of camera/NIR illuminator/ultrasonic sensor, and eyeball’s center is passing though the point of corneal SR because NIR illuminator produces the corneal SR. Therefore, the pupil-corneal SR vector can be calculated from *g* and *h*. The eyeball of *h*_1_, *g*_1_, *O*_1_, and *d*_1_ of Figure 5 represents the case before movement, and that of *h*_2_, *g*_2_, *O*_2_, and *d*_2_ of Figure 5 shows the case after movement.

In case of T_X movement (Figure 5a), we can obtain the Equations (3) and (4) based on the similarity property of triangle. From the Equations (3) and (4), we get the Equation (5). The approximate value of the pupil-corneal SR vector after T_X can be obtained as (*h*_2_ *+ g*_2_) as shown in Equation (6). This means that the pupil-corneal SR vector after T_X is same as that before T_X, and T_X does not change the original pupil-corneal SR vector:

(3) $$\left(d+r\right):H=r:h_{1},\;\left(d+r\right):H=r:h_{2}$$

(4) $$\left(d+r\right):G=r:g_{1},\;\left(d+r\right):G=r:g_{2}$$

(5) $$h_{1}=h_{2},\;g_{1}=g_{2}$$

(6) $$h_{2}+g_{2}=h_{1}+g_{1}$$

In case of T_Y movement (Figure 5b), we can obtain Equations (7) and (8) based on the similarity property of triangle. From Equations (7) and (8), we get Equation (9). The approximate value of the pupil-corneal SR vector after T_Y can be obtained as (*h*_2_ *– g*_2_) as shown in Equation (10). This means that the pupil-corneal SR vector after T_Y is same as that before T_Y, and T_Y does not change the original pupil-corneal SR vector:

(7) $$\left(d+r\right):H=r:h_{1},\;\left(d+r\right):\left(H+T_{y}\right)=r:h_{2}$$

(8) $$\left(d+r\right):G=r:g_{1},\;\left(d+r\right):(T_{y}-G)=r:g_{2}$$

(9) $$h_{2}=\frac{T_{y}r}{d+r}+h_{1},\;g_{2}=\frac{T_{y}r}{d+r}-g_{1}$$

(10) $$h_{2}-g_{2}=h_{1}+g_{1}$$

Using the same method, in Figure 5c, we can obtain the pupil-corneal SR vector after T_Z as (*h*_2_ *+ g*_2_) as shown in Equation (14) using Equations (11)–(13). As shown in Equation (14), the pupil-corneal SR vector after T_Z is changed according to the Z-distance ratio, considering *r* × ((*d*_1_ + *r*)/(*d*_2_ + *r*)) compared to the original pupil-corneal SR vector (*h*_1_ *+ g*_1_):

(11) $$\left(d_{1}+r\right):\;H=r:\;h_{1},\;\;\;\left(d_{2}+r\right):H=r:h_{2}$$

(12) $$\left(d_{1}+r\right):\;G=r:\;g_{1},\;\;\;\left(d_{2}+r\right):G=r:g_{2}$$

(13) $$h_{2}=\frac{d_{1}+r}{d_{2}+r}h_{1},\;\;\;\;\;\;g_{2}=\frac{d_{1}+r}{d_{2}+r}g_{1}$$

(14) $$h_{2}+g_{2}=\frac{d_{1}+r}{d_{2}+r}\left(h_{1}+g_{1}\right)$$

In case of R_X movement (Figure 5d), we can decompose the R_X movement into T_Z and T_Y movements. As shown in Figure 5b and Equation (10), T_Y does not change the original pupil-corneal SR vector. In addition, as shown in Figure 5c and Equation (14), the pupil-corneal SR vector after T_Z is changed according to the Z-distance ratio considering *r* × ((*d*_1_ + *r*)/(*d*_2_ + *r*)) compared to the original pupil-corneal SR vector (*h*_1_ *+ g*_1_). Therefore, we can obtain the pupil-corneal SR vector after R_X as shown in Equation (18), obtained using Equations (15)–(17):

(15) $$\left(d_{1}+r\right):\;H=r:\;h_{1},\;\;\;\left(d_{2}+r\right):\left(H+T_{y}\right)=r:h_{2}$$

(16) $$\left(d_{1}+r\right):\;G=r:\;g_{1},\;\;\;\;\left(d_{2}+r\right):(T_{y}-G)=r:g_{2}$$

(17) $$h_{2}=\frac{d_{1}+r}{d_{2}+r}h_{1}+\frac{T_{y}r}{d_{2}+r},\;\;\;\;\;\;g_{2}=\frac{T_{y}r}{d_{2}+r}-\frac{d_{1}+r}{d_{2}+r}g_{1}$$

(18) $$h_{2}-g_{2}=\frac{d_{1}+r}{d_{2}+r}\left(h_{1}+g_{1}\right)$$

In case of R_Y movement (Figure 5e), we can decompose R_Y movement into T_Z and T_X movements. Using a similar method for the case of R_X movement, T_X does not change the original pupil-corneal SR vector as shown in Figure 5a and Equation (6). In addition, the pupil-corneal SR vector after T_Z is changed according to the Z-distance ratio considering *r* × ((*d*_1_ + *r*)/(*d*_2_ + *r*)) compared to the original pupil-corneal SR vector (*h*_1_ *+ g*_1_). Therefore, we can obtain the pupil-corneal SR vector after R_Y as shown in Equation (22), obtained using Equations (19)–(21):

(19) $$\left(d_{1}+r\right):\;H=r:\;h_{1},\;\;\;\;\left(d_{2}+r\right):H=r:h_{2}$$

(20) $$\left(d_{1}+r\right):\;G=r:\;g_{1},\;\;\;\;\left(d_{2}+r\right):G=r:g_{2}$$

(21) $$h_{2}=\frac{d_{1}+r}{d_{2}+r}h_{1},\;\;\;\;\;\;g_{2}=\frac{d_{1}+r}{d_{2}+r}g_{1}$$

(22) $$h_{2}+g_{2}=\frac{d_{1}+r}{d_{2}+r}\left(h_{1}+g_{1}\right)$$

Finally, for the last case of R_Z movement (Figure 5f), we can decompose R_Z movement into T_X and T_Y movements. As explained before, T_X and T_Y do not change the original pupil-corneal SR vector. Therefore, the pupil-corneal SR vector after R_Z is same as that before R_Z, and R_Z does not change the original pupil-corneal SR vector as shown in Equation (26), obtained using Equations (23)–(25):

(23) $$\left(d+r\right):H=r:h_{1},\;\;\left(d+r\right):\left(H+T_{y}\right)=r:\;h_{2}$$

(24) $$\left(d+r\right):G=r:g_{1},\;\;\left(d+r\right):\left(G-T_{y}\right)=r:\;g_{2}$$

(25) $$h_{2}=\frac{T_{y}r}{d+r}+h_{1},\;\;\;\;\;\;g_{2}=g_{1}-\frac{T_{y}r}{d+r}$$

(26) $$h_{2}+g_{2}=h_{1}+g_{1}$$

To summarize, we can estimate that the change of the pupil-corneal SR vector is only affected by the change of Z-distance (Table 2).

**Table 2:** Change of the pupil-corneal SR vector according to head movements.

	X Axis	Y Axis	Z Axis
Translation	No change	No change	Change (Equation (14))
Rotation	Change (Equation (18))	Change (Equation (22))	No change

Although we assume that the Z-axis is orthogonal to the camera image plane as shown in Figure 4, we do not use the assumption that the head movement of user in the direction of Z-axis is limited only to the parallel direction of Z-axis. That is, although the head movement of user is not parallel to the direction of Z-axis, this case can be handled in our research by considering the combination of *T_z_* and *T_y_* (or *T_z_* and *T_x_*) as shown in Figure 5d,e.

### 2.3. Compensating the Head Movement

**Figure 6:**
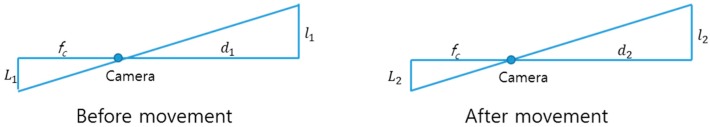
Camera perspective model.

The Equations (14), (18) and (22) are obtained in 3D space. Therefore, we should obtain the corresponding Equations in 2D image plane in order to compensate the head movement because the pupil-corneal SR vector can be measured in 2D captured image. For that, we apply a camera perspective model [24] as shown in Figure 6. *f_c_* is camera focal length, *d*_1_ or *d*_2_ is Z-distance, and *L*_1_ or *L*_2_ is the projection in image of *l*_1_ or *l*_2_ in 3D space. Then, we can get Equations (27) and (28). In Equations (14), (18) and (22), $h_{2}+g_{2}$ or $h_{2}-g_{2}$ is *l*_2_, and $h_{1}+g_{1}$ is *l*_1_. Therefore, we can obtain the Equation (29) using the Equations (14), (18), (22) and (28). Finally, the Equation (30) can be obtained, where *L*_1_ and *L*_2_ are the pupil-corneal SR vectors in the image before and after head movement, respectively. We can compensate the head movement using the Equation (30).

(27) $$l_{1}=\frac{L_{1}d_{1}}{f_{c}}\;,\;\;l_{2}=\frac{L_{2}d_{2}}{f_{c}}$$

(28) $$\frac{l_{2}}{l_{1}}=\frac{L_{2}d_{2}}{L_{1}d_{1}}$$

(29) $$\frac{l_{2}}{l_{1}}=\frac{L_{2}d_{2}}{L_{1}d_{1}}=\frac{d_{1}+r}{\;d_{2}+r}$$

(30) $$L_{2}=\frac{d_{1}+r}{\;d_{2}+r}*\frac{d_{1}}{d_{2}}*L_{1}$$

In order to compensate the head movement using Equation (30), we should know the Z-distances (*d*_1_ and *d*_2_) and the eyeball radius (*r*). We refer to Gullstrand’s eye model [28] for the eyeball radius. To get the Z-distance, *d_1_* and *d_2_*, we use a commercial ultrasonic sensor (SRF08) [29] set-up above the camera for gaze tracking. The sensor consists of two parts—transmitter & receiver— and a control board. The transmitter & receiver part is connected to the control board via an I2C interface. The control board is connected to a desktop computer via a universal serial bus (USB) 2.0 interface. Therefore, the Z-distance data measured by transmitter & receiver is continuously transmitted to the desktop computer via the control board at the frequency of 40 kHz. The maximum Z-distance which can be measured is limited to 6 m. The principle of the ultrasonic sensor is to measure the Z-distance between the transmitter & receiver and the object closest to the transmitter & receiver. Therefore, it is difficult to measure the accurate Z-distance between the ultrasonic sensor and the eye. Instead, it can measure the Z-distance between the ultrasonic sensor and another part of the face such as chin (or nose). In our research, the Z-distance between the chin (or nose) and eye is much smaller (2~3 cm) compared to the Z-distance (70~80 cm) between the chin (or nose) and ultrasonic sensor. Therefore, we can use the assumption that the Z-distances of the chin (or nose) and eye to the sensor are similar. In addition, the ultrasonic sensor can measure the Z-distance, even when a user wears glasses.

However, the stability for measuring an accurate Z-distance with the ultrasonic sensor is not high because the Z-distance can change even while the user’s head rotation is not changed in the Z-distance. To overcome this problem, we use the inter-distance between two eyes (pupil centers) (in the 3D space) which is measured during the initial stage of user calibration. The Z axis of the head in Figure 4 is usually oriented towards the ultrasonic sensor when a user gazes at the center-lower position of the monitor (close to the position of the ultrasonic sensor) during the initial stage of user calibration. Then, we can obtain the Z-distance (*d*) using the ultrasonic sensor and assume that *d* is approximately the distance between the camera and user’s eye because the camera is close to the ultrasonic sensor. With the known camera focal length (*f_c_*) (which is obtained by initial camera calibration), the measured Z-distance (*d*), and the inter-distance between two pupil centers (in the image) (*L*), we can obtain the actual inter-distance between two pupil centers (in the 3D space) (*l*) using the equation (*l* = (*d*·*L*)/*f_c_*) based on camera perspective model of Figure 6.

This actual inter-distance between two pupil centers (in the 3D space) (*l*) is not changed even when head movement occurs. Therefore, since we know the inter-distance between two eyes (in the 3D space) (*l*_2_ (=*l*) of Figure 6) and the changed inter-distance in the camera image after head movement (*L*_2_ of Figure 6) with the camera focal length (*f_c_*), we can estimate the changed Z-distance after head movement (*d*_2_ of Figure 6) using the equation (*d_2_* = (*l*_2_·*f*_c_)/*L*_2_). 

**Figure 7:**
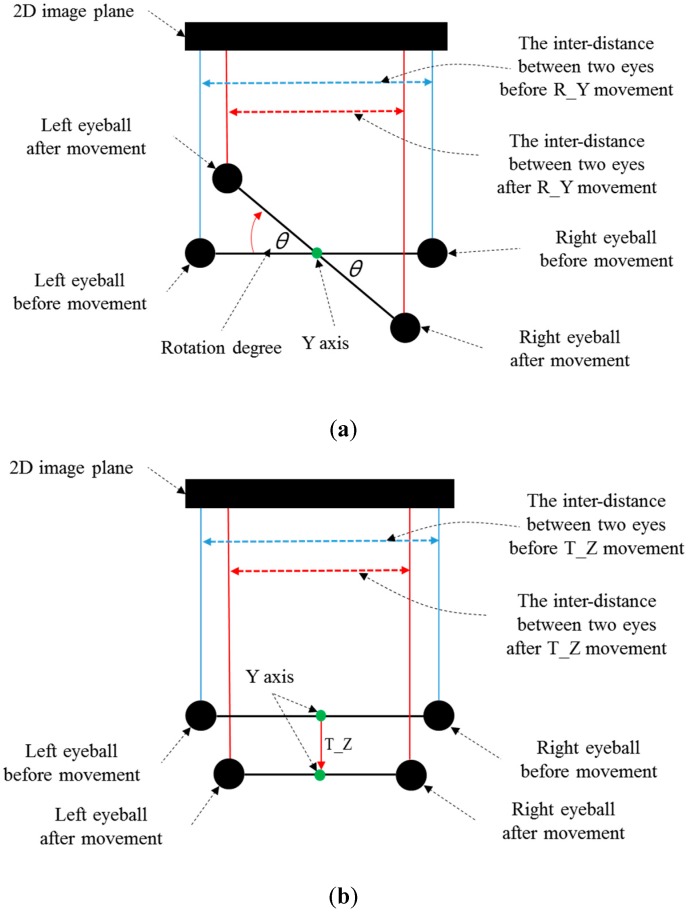
Difference of the inter-distance between two eyes before and after (**a**) R_Y movement (**b**) T_Z movement (top view).

In general, there are many individual variances of the inter-distance (*l*) between two eyes (pupil centers) (in the 3D space). Therefore, if we use the average value (calculated from many people) of *l* for measuring the Z-distance, the measurement error of Z-distance is much higher than that by our method. Experimental results with 10 people showed that the measurement error of the Z-distance by using the average value of *l* (calculated from many people) was about 3.5 cm, while that measured by our method was less than 1 cm. However, in the case of R_Y movement, the inter-distance between two eyes and the corresponding Z-distance are measured incorrectly. This is caused by the mapping of the 3D space object into 2D. As shown in Figure 7a,b, the inter-distance between two eyes in the 2D image plane becomes smaller in both cases of R_Y and T_Z. Although the pupil-corneal SR vector is changed, and it should be compensated in case of R_Y as shown in Equation (22), our system does not need to perform the compensation. The reason for this is our use of binocular gaze tracking. As shown in Figure 7a, the left eye is closer to the monitor whereas the right one becomes farther away in the case of R_Y. Based on the Equations (22) and (30), *d_2_* is smaller in the case of the left eye whereas *d_2_* is larger in the case of the right one. Because our system uses binocular gaze tracking by obtaining the average gaze position of two eyes, the head movement by R_Y does not need to be compensated. However, in case of T_Z, the head movement must be compensated as shown in Table 2. For that, our system should discriminate the R_Y and T_Z movement, which is achieved as explained below.

**Figure 8:**
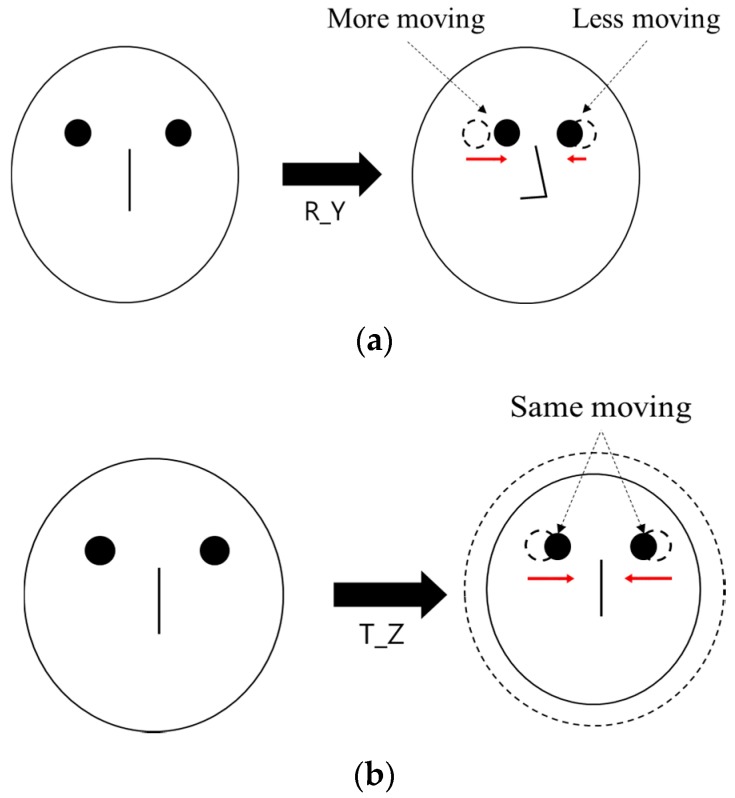
Difference of the movement of both eyes on horizontal axis in case of (**a**) R_Y movement (**b**) T_Z movement.

When R_Y movement occurs, the amount of movement of both eyes on the horizontal axis is different from each other as shown in Figure 8a, whereas it is almost similar in case of T_Z movement as shown in Figure 8b. Based on this, our system discriminates the case of R_Y movement from that of T_Z movement, and compensates the user’s head movement by compensating the pupil-corneal SR vector in the image based on Equation (30). 

## 3. Experimental Results

### 3.1. Comparisons of Gaze Tracking Errors without and with Our Compensation Methods

To compare the gaze accuracies with or without the proposed head movement compensation, we performed experiments with data from ten subjects. In most previous research on gaze tracking, experiments were performed with less than ten subjects. In the research of [15,16,19,20], and [23], the number of subjects participating in the experiments were seven, nine, two, three and six subjects, respectively. Each subject moved his or her head according to two movements (translation and rotation), three axes (X, Y, and Z), and two directions (plus and minus of the translation, clockwise and counterclockwise rotation). The proposed algorithm was executed on a desktop computer having a CPU configuration of 3.47 GHz (Intel (R) Core (TM) i7 CPU X 990) and 12 GB RAM with a monitor of diagonal size of about 48.3 cm and having 1280 × 1024 pixel resolution. As a gaze tracking camera, a conventional web-camera was used (Logitech C600 [30]). The viewing angle of camera lens is 10° (−5°–+5°). Therefore, the size of the viewport at the typical Z-distance (about 70 cm) is approximately 12.2 cm (=2 × 70 × tan5°). The viewing angle of original camera lens is much larger than 10 degrees. However, with this lens, the eye region becomes too small in the captured image, which can degrade the accuracy of gaze detection. Therefore, we replace the original lens by a zoom lens whose viewing angle is 10° (−5°–+5°) (whose focal length is 22 mm). This lens is made by other Korean optical lens company [31]. Using this lens, we can capture the magnified eye image.

In order to capture images at fast speed (30 frames/s), an image of 800 × 600 pixels was acquired, and was re-sized to 1600 × 1200 pixels by bi-linear interpolation. Although the image of 1600 × 1200 pixels can be captured by the camera, the data size of this image is four times larger than that of the image of 800 × 600 pixels. Therefore, the capturing speed becomes slower (7–8 frames/s (=30/4 frames/s)) due to the bandwidth limitation of the data transfer from the camera to computer, and the fast movement of user’s head and eye cannot be captured by the camera, consequently. In addition, through the resizing by bi-linear interpolation, additional pixel information of eye region can be obtained, through which more accurate detection of pupil and corneal SR can be possible, and the consequent accuracy of gaze detection becomes higher. 

**Figure 9:**
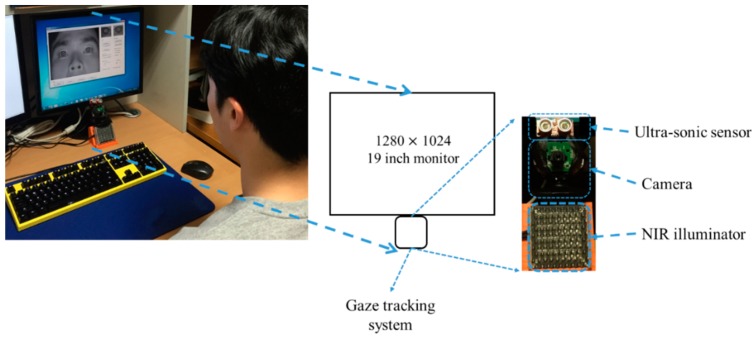
Experimental environment.

**Figure 10:**
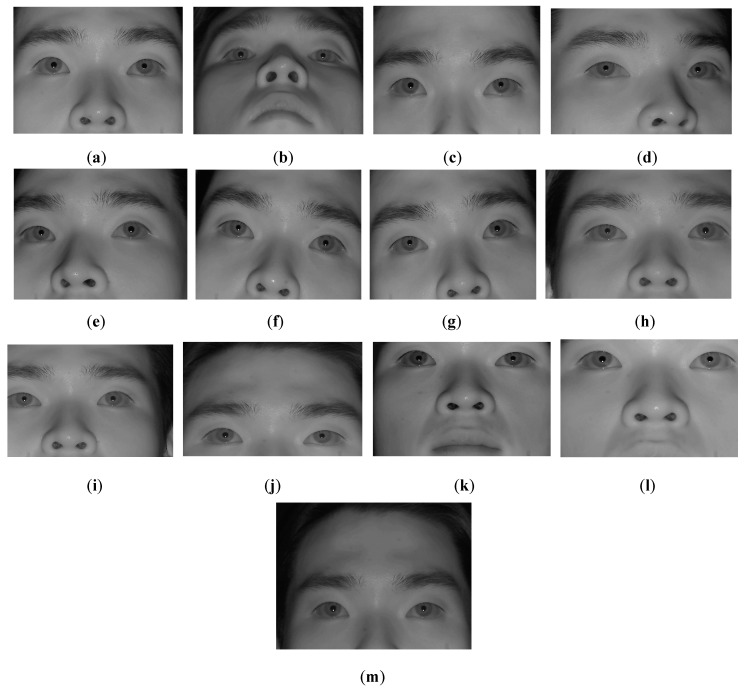
Examples of images according to each head movement: (**a**) No head movement, (**b**) R_X–, (**c**) R_X+, (**d**) R_Y–, (**e**) R_Y+, (**f**) R_Z–, (**g**) R_Z+, (**h**) T_X–, (**i**) T_X+, (**j**) T_Y–, (**k**) T_Y+, (**l**) T_Z–, and (**m**) T_Z+.

To get the NIR image, the NIR cutting filter inside the web-camera is replaced by a long pass filter (Wratten Filter No. 89B) which can pass the NIR light with a wavelength longer than 700 nm [32]. The proposed algorithm was implemented with C++ language using Microsoft Foundation Class (MFC) and OpenCV library (ver. 2.4.5 [33]). Figure 9 shows the experimental environment. In Figure 10, we show the examples of experimental images according to each head movement.

After each user performed the user dependent calibration by gazing at nine reference points on the monitor (calibration stage), the gaze tracking errors with or without the proposed method were measured (with the data which are obtained when each user gazes at 20 reference points) according to the head movements (testing stage). The results are shown in Table 3. Gaze tracking error is measured as the angular difference (error) between the calculated gaze vector and the vector to the reference (ground-truth) point, and STD means the standard deviation of gaze tracking error. Therefore, a smaller error means a higher gaze detection accuracy. As shown in Table 3, the gaze detection errors achieved with our proposed method are lower than those achieved without our method, in all cases of head movement. In addition, as shown in Table 3, the gaze detection error with our method is less than 0.9° in both cases of no head movement and head movement.

**Table 3:** Average gaze detection errors of ten subjects with or without our method (unit: °).

Kinds of Head Movement	Average Gaze Detection Error (STD)	
	Without our Method	With our Method
No head movement	0.58 (0.06)	
R_X–	2.32 (0.42)	0.81 (0.17)
R_X+	2.69 (0.35)	0.70 (0.19)
R_Y–	0.71 (0.18)	0.69 (0.13)
R_Y+	0.70 (0.09)	0.65 (0.11)
R_Z–	0.74 (0.16)	0.71 (0.70)
R_Z+	0.70 (0.14)	0.69 (0.16)
T_X–	0.73 (0.12)	0.67 (0.10)
T_X+	0.71 (0.13)	0.61 (0.10)
T_Y–	0.68 (0.13)	0.65 (0.17)
T_Y+	0.68 (0.20)	0.63 (0.10)
T_Z–	8.43 (1.32)	0.74 (0.17)
T_Z+	3.82 (0.98)	0.77 (0.12)
Average	1.81 (0.11)	0.69 (0.06)

**Table 4:** Individual gaze detection errors with or without our method (unit: °).

Subjects	Average Gaze Detection Error	
	Without our Method	With our Method
1	1.71	0.67
2	1.71	0.59
3	1.81	0.59
4	1.89	0.74
5	2.04	0.75
6	1.80	0.64
7	1.80	0.77
8	1.64	0.72
9	1.88	0.75
10	1.79	0.7
Average	1.81	0.69

In Table 4, we show the individual gaze detection errors with and without our method. As shown in Table 4, the average gaze detection error achieved with our method is lower than the error achieved without our method, in all the subjects.

In order to prove that the average gaze detection error achieved using our method is statistically lower than the error achieved without our method, we performed a t-test [34]. When the t-test was performed using two independent samples (two gaze detection errors without our method (1.81° with STD of 0.11° as shown in Table 3) and with our methods (0.69° with STD of 0.06° as shown in Table 3)), the calculated p-value was 4.43105 × 10^−16^, which was smaller than the 99% (0.01) significance level. Then, the null-hypothesis for the t-test (there is no difference between the two independent samples) is rejected based on the p-value. Therefore, we can conclude that there is a significant difference between the average gaze detection errors with and without our method at the significance level of 99%.

We performed Cohen’s d analysis as a next step, by which the size of the difference between the two groups can be indicated using the effect size [35]. In general, Cohen’s d is determined as small at about 0.2–0.3, medium at about 0.5, and large at or higher than 0.8. The calculated Cohen’s d is 12.1765, and we can conclude that the difference between the average gaze detection errors without and with our method is large. From the p-value and Cohen’s d, we can conclude that there is a significant difference in the average gaze detection errors with or without our method.

In the next experiment, we measured the processing time required by our method. The total processing time taken by our method is 15.55 ms per frame, and we can know that our system can be operated at the speed of about 64.3 frames/s. For experiments, each participant moved his or her head within the range of head movement as shown in Table 5.

**Table 5:** Range of head movements allowed in our system.

Kinds of Head Movement	Range
R_X+	25°
R_X–	25°
R_Y+	25°
R_Y–	25°
R_Z+	25°
R_Z–	25°
T_X+	2.5 cm
T_X–	2.5 cm
T_Y+	3 cm
T_Y–	3 cm
T_Z+	10 cm
T_Z–	10 cm

### 3.2. Comparisons of Gaze Tracking Errors with Our Method and Commercial System

In the next experiment, we compared the gaze detection errors by our method with that of a commercial gaze tracking system. As an example, a TheEyeTribe commercial system is used [14]. To compare the gaze accuracies, we performed experiments with data from ten subjects like the experiments of Section 3.1. 

Each subject moved his or her head according to two movements (translation and rotation), three axes (X, Y, and Z), and two directions (plus and minus of the translation, clockwise and counter-clockwise of the rotation). Like the experiments of Section 3.1, the proposed algorithm was executed on a desktop computer having a CPU configuration of 3.47 GHz (Intel (R) Core (TM) i7 CPU X 990) and 12 GB RAM with a monitor of diagonal size of about 48.3 cm and having 1280 × 1024 pixel resolution. Experimental results showed that our system produce lower gaze detection errors in all cases of head movements than the commercial system (TheEyeTribe) as shown in Table 6.

**Table 6:** Comparisons of gaze detection errors with commercial system (TheEyeTribe) (unit: %).

Head Movement	Gaze Detection Error (STD)	
	Our Method	Commercial System
R_X+	0.70 (0.19)	1.61 (0.73)
R_X–	0.81 (0.17)	1.36 (0.50)
R_Y+	0.65 (0.11)	1.69 (0.24)
R_Y–	0.69 (0.13)	1.87 (0.65)
R_Z+	0.69 (0.16)	0.79 (0.14)
R_Z–	0.71 (0.15)	0.69 (0.02)
T_X+	0.61 (0.10)	1.04 (0.64)
T_X–	0.67 (0.10)	1.08 (0.25)
T_Y+	0.63 (0.10)	1.05 (0.26)
T_Y–	0.65 (0.17)	0.70 (0.12)
T_Z+	0.77 (0.12)	1.42 (0.17)
T_Z–	0.74 (0.17)	4.53 (0.43)
Average	0.69 (0.14)	1.49 (0.35)

**Figure 11:**
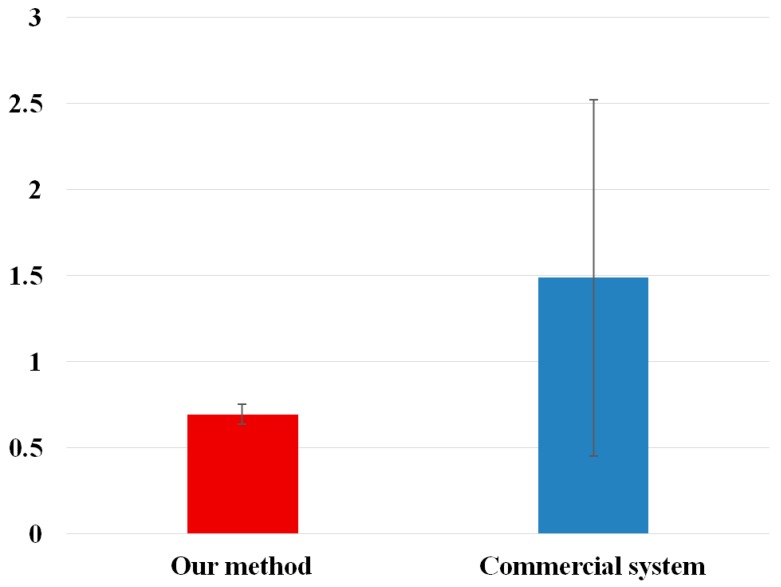
T-test of average gaze detection errors by our method and a commercial system (TheEyeTribe).

In Figure 11, we show the t-test, and the analysis of Cohen’s d. The measured p-value is 0.014569, which was smaller than the 95% (0.05) significance level, and we can conclude that there is a significant difference in the average gaze detection errors achieved using our method and those achieved using commercial system. In addition, the measured Cohen’s d is 1.0826, and we can conclude that the difference between the average gaze detection errors achieved using our method and commercial system is large.

In the last experiment, we compared the gaze detection errors by our method with that of another commercial gaze tracking system. As an example, the commercial system (Tobii EyeX) is used [36]. To compare the gaze accuracies, we performed experiments with data from ten subjects like the experiments of Section 3.1. Each subject moved his or her head according to two movements (translation and rotation), three axes (X, Y, and Z), and two directions (plus and minus of the translation, clockwise and counter-clockwise of the rotation). 

Like the experiments of Section 3.1, the proposed algorithm was executed on a desktop computer having a CPU configuration of 3.47 GHz (Intel (R) Core (TM) i7 CPU X 990) and 12 GB RAM with a 19-inch monitor having 1280 × 1024 pixel resolution. Experimental results showed that our system produce lower gaze detection errors in all cases of head movements than the commercial system (Tobii EyeX) as shown in Table 7. 

**Table 7:** Comparisons of gaze detection accuracies with commercial system (Tobii EyeX) (unit: %).

Head Movement	Gaze Detection Error (STD)	
	Our Method	Commercial System
R_X+	0.70 (0.19)	0.59 (0.24)
R_X–	0.81 (0.17)	0.99 (0.52)
R_Y+	0.65 (0.11)	0.69 (0.55)
R_Y–	0.69 (0.13)	0.77 (0.37)
R_Z+	0.69 (0.16)	0.92 (0.50)
R_Z–	0.71 (0.15)	0.92 (0.42)
T_X+	0.61 (0.10)	1.01 (0.35)
T_X–	0.67 (0.10)	0.90 (0.14)
T_Y+	0.63 (0.10)	1.17 (0.53)
T_Y–	0.65 (0.17)	0.67 (0.24)
T_Z+	0.77 (0.12)	0.77 (0.29)
T_Z–	0.74 (0.17)	0.87 (0.23)
Average	0.69 (0.14)	0.86 (0.37)

**Figure 12:**
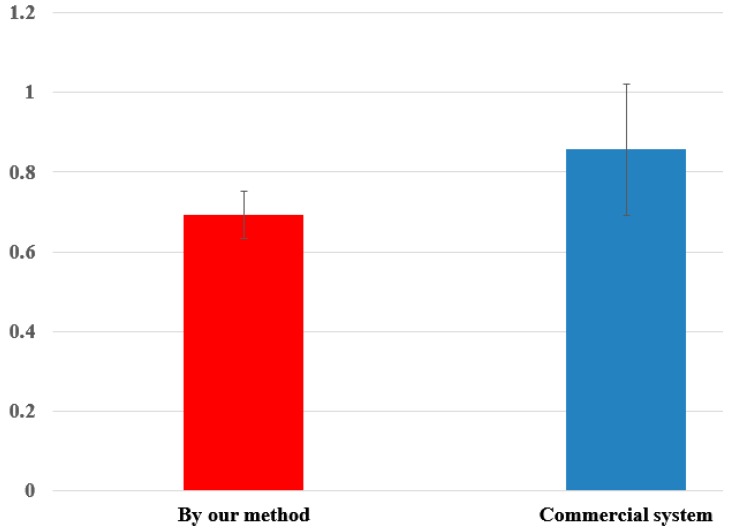
T-test of average gaze detection accuracies by our method and commercial system (Tobii EyeX).

In Figure 12, we show the t-test, and the analysis of Cohen’s d. The measured p-value is 0.005853, which was smaller than the 99% (0.01) significance level, and we can conclude that there is a significant difference in the average gaze detection accuracies achieved using our method and those achieved using commercial systems. In addition, the measured Cohen’s d is 1.32538, and we can conclude that the difference between the average gaze detection accuracies achieved using our method and commercial system is large.

The reason why we selected two commercial systems such as TheEyeTribe [14] and Tobii EyeX [36] is that these systems have been the most widely used as commercial gaze detection systems due to their advantages of high performance and low cost. When people use a gaze tracking system, it is often the case that user’s head moves from the position where user’s calibration is performed. This causes the degradation of gaze detection accuracy in most gaze tracking systems. To overcome this problem, our head movement compensation method is proposed, and our method can guarantee the high accuracy of gaze detection even while user’s natural head movement occurs after initial user calibration. These statements are confirmed through the experimental results of Table 3, Table 4, Table 6 and Table 7 and Figure 11 and Figure 12.

In [37], Ishima and Ebisawa proposed an eye tracking method using an ultrasonic sensor for head free gaze detection. However, their method requires users to wear a head-mounted device including three ultrasonic transmitters. In addition, three ultrasonic receivers should be attached on known (three) positions around the monitor frame. By using the head-mounted device and three ultrasonic transmitters & receivers, user convenience is lower and the size of the system becomes bigger. In addition, because of the uss of the three ultrasonic receivers at predetermined positions around the monitor frame, additional time for setting up the system is required when a different monitor is used. Also they did not show the accuracy of gaze detection, but rather present the robustness of continuously capturing eye images with their system. Our system uses only one ultrasonic sensor (transmitter & receiver) as shown in Figure 9. By using one sensor, the size of the system is very small and the set-up of the system is easy, irrespective of the kind of monitor. In addition, our system does not require users to wear any device, which can enhance the users’ convenience. Different from [37], we showed that our system can guarantee the high accuracy of gaze detection irrespective of a user’s natural head movement after the initial user calibration. 

Our system also has cost advantages compared to the two commercial systems. Our system is composed of one web-camera (less than $20), one ultrasonic sensor (less than $20), and NIR illuminator with filter (less than $20). Therefore, the total cost of our system is less than $60, which is cheaper than the two commercial systems (TheEyeTribe [14] and Tobii EyeX [36]) whose cost are about $100–$150, respectively.

## 4. Conclusions

In our research, we propose a gaze tracking method using an ultrasonic sensor which is robust to the natural head movement of a user. Through mathematical analyses of the change of relative positions between the PC and corneal SR due to various head movements, we find that only the head movement (causing the change of Z-distance between the camera and user’s eye) can affect the gaze detection accuracy. We measure the change of Z-distance of a user’s eye using a small sized ultrasonic sensor, which does not require any complicated camera calibration and its Z-distance measurement is much faster than possible with stereo cameras. The accuracy of the measured Z-distance by the ultrasonic sensor was enhanced by using the change of inter-distance of two eyes in the images. By using the information of eye movement change for both eyes in X-axis of the image, we can discriminate R_Y from T_Z. Experimental results show that the gaze tracking system with our compensation method is more robust to natural head movements than the systems without our method and commercial systems. The results are proved by t-test and the analysis of effect size.

In the future, we would measure the performance of our system in more varied environments. In addition, we would research a method for enhancing the accuracy of gaze detection systems, which are robust to user’s head movement by combining the proposed method with the train-based estimator of Z-distance.
